# Soil health indicators for Central Washington orchards

**DOI:** 10.1371/journal.pone.0258991

**Published:** 2021-10-28

**Authors:** Sara Tianna DuPont, Lee Kalcsits, Clark Kogan

**Affiliations:** 1 Tree Fruit Research and Extension Center, Washington State University, Wenatchee, Washington, United States of America; 2 Department of Mathematics and Statistics, Washington State University, Pullman, Washington, United States of America; Carnegie Mellon University, UNITED STATES

## Abstract

Soil health assessment can be a critical soil testing tool that includes biological and physical indicators of soil function related to crop and environmental health. Soil health indicator minimum data sets should be regional and management goal specific. The objective of this study was to initiate the steps to develop a soil assessment tool for irrigated orchard soils in Central Washington, United States including defining objectives, gathering baseline data and selecting target indicators. This study measured twenty-one biological, physical and chemical properties of soils in irrigated Central Washington apple orchards including indicators of water availability, root health, fertility, and biological activity. Soil factors were related to fruit yield and quality. Principal components and nonlinear Bayesian modeling were used to explore the relationship between soil health indicators and yield. Soil indicators measurements in Washington state orchards varied widely but generally had lower organic matter, available water capacity, wet aggregate stability and higher percent sand than in other regions. Linear mixed effects models for available water capacity and percent sand showed significant effects on yield, and models for root health ratings and *Pratylenchus* nematodes had moderate effects. The minimum dataset of soil health indicators for Central Washington orchards should include measurements of water availability (available water capacity, percent sand) and of root health (bean root health rating, *Pratylenchus* nematodes) in addition to standard fertility indicators to meet stakeholder management goals.

## Introduction

Soil health refers to the ability of soil to perform key ecosystem functions: sustaining plant growth, minimizing erosion, regulating water flow, and filtering toxic materials [1–5]. A soil health framework allows us to assess not only the immediate relationship between soil and yield, but also track long-term impacts of soil erosion on crop productivity. The terms soil quality and soil health are generally interchangeable in the literature and can be considered equivalent [6]. In this paper we use the term soil health.

Soil health assessment strategies have been developed to measure indicators of key soil functions. Soil health indices generally include the physical, biological and chemical properties of soil related to ecosystem processes which are sensitive to management. Researchers often start with a minimum data set (MDS) of selected indicators and use multiple methods to calculate indices based on targeted ecosystem functions [3, 7–9]. For example, the Soil Management Assessment Framework allows users to select a set of indicators based on management objectives out of eighty-one potential indicators [10, 11]. The Comprehensive Assessment of Soil Health (CASH) includes a suite of fifteen biological, physical and chemical property indicators chosen based on sensitivity to changes in management practices, ability to represent important agronomic and environmental processes, consistency, reproducibility, cost and ease of sampling, cost of analysis and ease of interpretation for users, and is interpreted through scoring functions [4, 10, 12–14]. The Haney test and ‘Soil health tool’ calculates a soil health score based on microbial activity, readily available carbon sources for microbial respiration, and the ratio of carbon to nitrogen in the sample [15–17]. The goal of this test is to better account for microbially available nutrients and reduce unnecessary fertilizer use. Australian and New Zealand groups use national and regional survey data to create benchmarks and threshold values [18, 19]. Many methods have been used in Europe, focusing on biodiversity [20] or target values and median values based on large surveys [21].

Appropriateness of soil health assessment tools varies by region, cropping system and management goals. However currently, few soil health assessment tools have been evaluated in perennial crops in the irrigated region of Western North America [22]. Assessment tools have generally been tested for annual crops and in the rainfed Mid-west, Northeast and Mid-Atlantic [5, 17, 23–27] with only a few tools assessed in irrigated annual crops [28]. To our knowledge the only soil health assessment tool for perennial crops in the irrigated Western United States is from Glover, Reganold [29] adapted from the approach of Karlen and Stott [1].

Relatively few studies evaluate the relationship between soil health indicators and crop productivity. van Es and Karlen [30] reported that soil health indicators active carbon, protein, respiration and Mn were correlated with corn and soybean yield. Haney, Haney [17] found that even soils with a low soil health score were capable of growing corn efficiently. Stine and Weil [31] found that macroaggregate stability and soil C (particularly active carbon) were highly correlated with crop productivity. In a review of thirteen studies across seventy-six sites Hurisso, Culman [32] found that active carbon (permanganate oxidizable carbon) predicted agronomic performance. SOC and wet aggregate stability explained more than thirty five percent of grain yield variability in a study working to develop soil quality indices in Ohio [33].

In perennial crops such as apples, pears and cherries both crop productivity and quality are key management goals. In some cases, high yield does not result in large numbers of fruit sold due to the relationship between cropload and fruit quality [34]. While a number of studies have used yield in the creation of soil quality indices, only a very few studies have looked at the relationship between crop quality and soil health characteristics. For example, Rekik, van Es [35] found a negative correlation between available water capacity, organic matter and active carbon with coffee quality. To our knowledge this paper is the first to study soil health assessment tools for irrigated orchards in the Western United States which looks at the relationship between indicators and stakeholder management goals of yield and quality.

This paper initiates the steps to develop a soil assessment tool suggested by Bunemann, Bongiorno [6] for irrigated orchard soils in the western United States: 1) define objectives and identify target users 2) establish reference baseline data 3) select indicators for targeted soil functions and 4) create interactive soil quality assessment tools. The stakeholder group of orchardists and managers in Washington state identified fruit yield and quality as the primary functions of interest [36]. A survey of 97 apple orchard plots was conducted between 2015 and 2019 in Washington State measuring 21 biological, chemical and physical properties of soil. A combination of exploratory plotting and linear mixed effects models were used to identify indicators with linkages to the soil function of interest and productivity. A combination of principal components and Bayesian models were used to explore a biologically reasonable relationship between soil indicators and productivity. An assessment tool is proposed including indicators selected by their relationship to apple productivity in addition to indicators related to ecosystem services established by the literature.

## Materials and methods

### Design and sampling

A total of 97 apple (*Malus domestica)* orchard fields were sampled (S1 Table, S1 Fig). Of these plots there were 30 matched pairs (60 plots) on the same or similar soil type with matching cultivar/rootstock, tree age and training system type. One field in each pair was high performing and the other underperforming based on grower description. A subset of 32 plots (16 matched pairs) were sampled for fruit yield and fruit quality.

Soil samples were collected as 50 to 100-soil core composite samples (2.5 cm core) to a depth of 20 cm in the tree root zone (15–60 cm from trunk) where 10 to 20 random samples were taken in each of five locations of the 2 to 16 hectare field. Four 100 cm^3^ intact soil cores were used for bulk density analysis. Five 120 cm^3^ intact cores were taken for micro-arthropod analysis. All soil sampling was conducted during June and July after soils had warmed (>13 °C) and 1 to 3 months before apple harvest.

Water supply for each orchard site was determined using a grower survey of irrigation practices and on-site irrigation measurements and demand was estimated from evapotranspiration estimates based on meteorological data from the nearest weather station [37]. On site irrigation measurements included measurements of water volume per emitter per hour at ten locations within a block which was then multiplied by emitters per acre. Fields with insufficient irrigation were not included in matched plot analysis.

Fruit yield and quality were determined by collecting grower reported packing house yield data for the previous two to four years where available. For orchards where packing house data was not collected, a subset of five representative trees were selected for each orchard. At harvest, fruit per tree were counted and 20 fruit per tree collected to determine mean fruit weight and to estimate total yield. Yield was normalized to a 0–100 scale (percent yield goal) where the reported yield was divided by the yield goal identified by the grower in order to account for the yield potential of individual varieties and growing systems.

### Fruit quality evaluation

To assess the proportion of high-quality fruit free of sunburn, bitter pit, or poor color, 60 fruit were collected from the three representative trees in each orchard. Fruit quality assessments included sunburn analysis following the Washington Tree Fruit Research Commission sunburn scale for bi-color fruit based on [38]. Red overcolor was graded based on <25% coverage, 25–50% coverage, 50–75% coverage, or 75–100% coverage. Bitter pit, lenticel breakdown or other external disorders were also assessed on all fruit. If fruit contained less than 50% red over color, bitter pit, or sunburn incidence that was greater than class two (YII), fruit was classified as a cull. From this, packout percentage and total packout (packed boxes per acre) was calculated for each orchard.

### Quantification of soil health indicators

The following procedures were performed by the Cornell Soil Health Lab per [39]. Texture was determined using the rapid soil texture procedure [40]. Available water capacity (AWC) was measured using pressure chambers and ceramic plates with a known porosity [41]. Wet aggregate stability (WAS) was measured using the rainfall simulator method [41]. Autoclaved-citrate extractable protein (ACE) was measured where proteins are extracted using sodium citrate solution [42]. Potentially mineralizable N (PMN) was determined using a saturated, anaerobic incubation [43]. Organic matter (OM) was determined by the loss on ignition method [44]. Permanganate oxidizable carbon (POXC) was measured using the permanganate oxidation method [45]. Respiration was determined from CO_2_ release during a four day incubation of air dried and then rewetted soil [17, 46].

Nutrient analysis was performed on dry samples following Western States methods: Olsen P extraction, and sodium bicarbonate K extraction [44] at Spectrum Analytic, Ohio.

Water infiltration and penetration resistance (PR) were measured in the field. Water infiltration was measured by inserting five 15 cm diameter rings into the soil in the weed free strip beneath the trees to a depth of 7 cm [47]. Rings were lined with plastic and filled with water to a depth of 2.5 cm. The plastic lining was removed and the time for water to infiltrate recorded to an upper limit of five minutes. PR an indicator of surface and subsurface compaction, was measured using a penetrometer in pounds per square inch (psi) (Dicky-john Corp, Auburn, IL) at field capacity. Slow even pressure was applied. The highest pressure reading was recorded for the surface 0–15 cm and proximate 15–45 cm.

Nematode populations were assessed from 250 cm^3^ samples that were extracted using a combination of decanting, sieving and Baermann funnel methods [48]. Nematodes were counted using a dissecting microscope and the first 200 nematodes encountered in the sample identified at 200X to 400X to genus or family. Soil food web indices were calculated after Ferris, Bongers [49] with trophic groups asigned per Yeates, Wardle [48] and colonizer-persister groups (cp) based on Bongers [50] and Bongers and Bongers [51]. Soil-dwelling micro-arthropod populations were measured using 120 cm^3^ intact cores that were stored at 1 °C and extracted within 10 days of collection using a modified Berlese-Tullegren funnel [52].

The apple root health rating was adapted from Laurent, Merwin [53], where apple seedling growth was used as the root health indicator. To prepare the soil, one half of each soil sample was moistened and pasteurized twice, at 80 °C for eight hours. Uniform six to eight-week old seedlings with six to eight leaves were selected to transplant into either pasteurized (N = 3) or un-pasteurized (N = 3) soil from each study site (160 mL conetainer pots). After eight weeks, seedling growth was assessed by measuring the length of the main stem and weighing dry biomass. A bean root health rating was conducted per Abawi, Ludwig [54] where bean seeds were planted in each of four 160 mL conetainer pots. After five weeks of greenhouse growth plant roots were hand washed and rated on a 1–9 scale where low ratings indicate a lack of, and higher ratings indicate strong presence of visible disease symptoms. Bulk density (w/v) was measured for four 100 cm^3^ dry, intact soil cores [55].

### Soil health indicator scoring functions

Soil health indicator scoring functions were calculated according to Fine, van Es [13] when available and appropriate in order to compare values measured in this study to those of large datasets collected in Mid-Atlantic, Midwest and Northeast regions. New scoring functions were calculated for macronutrients, pH, nematodes and percent sand based on optimum ranges for apples, and for water infiltration and apple bioassays using means and standard errors from the current dataset.

Soil organic matter, active carbon, aggregate stability, ace protein, potentially mineralizable N, bean bioassay and compaction were calculated as a scoring function of the normal distribution where *p* is the probability (between 0 and 1) that a measured value *x* will fall at a given position in the interval (+∞, -∞), and μ is the indicator mean and σ is the standard deviation following Fine *et al*. (2017) $p=f\left(x,\mu ,\sigma \right)=\frac{1}{\sigma \sqrt{2\pi }}\int_{-\infty }^{+\infty }e\frac{-{\left(x-\mu \right)}^{2}}{{2\sigma }^{2}}dx$. Means and standard deviations were derived from Fine 2017 based on 5,767 samples. Water infiltration and the apple bioassays were calculated using the above scoring function of the normal distribution with means and standard errors derived from the current dataset.

Nematode Structure Index and Basal Index were calculated according to Yeates, Bongers [56] Bongers [50], and Bongers and Bongers [51]. Bulk density (BD) ratings were calculated according to Raiesi [57] $Y=\frac{\text{max}x}{{1+(\frac{x}{mean\;x})}^{slope}}$, where the mean of x = 1.5, max x = 1, the slope = 2.5 and less than 1.5 g/cm^3^ is desirable. pH ratings were calculated based on a normal curve where the optimal range is 6 to 6.5, $y=\frac{1}{\sigma \sqrt{2\pi }}\times e^{{\left(-\frac{1}{2}(\frac{x-\mu }{\sigma }\right)}^{2})}$ where μ = mean (6.25), $\sigma =\frac{shoulder-min}{3}(\frac{6-2.1}{3})$, *e* = 2.7183 (constant). Lesion nematode scores were calculated according to Glover, Reganold [29] y=1-1[1+(LB-LTx-LT)^2S(LB+x-2×LT)]×100 where LB = lower baseline (90), LT = lower threshold (0.1) based on thresholds where 20–70 lesion nematodes per 500 g may cause crop damage and 80+ will likely cause damage in young trees. Scores for sand were also calculated according to Glover, Reganold [29] with both upper and lower optimum values ifY≤μ,y=1[1+(LB-LTx-LE)^2S(LB+x-2×LT)] ifY≥μ,y=1[1+(UB-UTx-UE)^-2S(UB+x-2×UT)] where LB = lower baseline (20), LT = lower threshold (5), LE = lower extent (lowest value) (0), UT = upper threshold (85), UB = upper baseline (70), UE = upper extent (highest value) (100). Phosphorus ratings were calculated using the above curve where UT = 200, UB = 100, LB = 7, LT = 1, slope = 0.428, -0.428. Potassium scores were calculated based on an optimum range of 100 to 250 ppm where the slope for values less than 100 is 3.1416 and for values greater than 250 is 0.428 where the rise of the curve was y=1[1+(LB-LTx-LE)^2S(LB+x-2×LT)] and downward slope was *y* = *L* × *fe* × 250 where $L=\frac{1}{\sqrt{2\pi }}$ and $fe=e^{-\frac{upper}{lower}}$ and *e* = 2.7183.

### Statistical methods

A linear regression model was conducted to establish the overall capacity to predict change in yield within blocks. A lasso penalization on the regression parameters was employed due to the large number of variables and the small sample size, and the lasso parameter was chosen using predicted residual sum of squares cross validation. Linear mixed effects models were fit to characterize the marginal association between yield (percent yield goal) and each of the selected soil health factors which showed strong trends in exploratory analysis. Each model included a piece-wise linear function (threshold) of the soil health factor as a covariate and block as a random effect. The mixed effects modeling used pre-determined thresholds and identified the magnitude of a linear effect after passing the threshold.

We further considered a nonlinear modeling approach that allowed the effect of a soil health variable to depend on the values of other soil health variables. This model allowed for instance, moderate changes from normal in nutrient concentrations to have little to no effect on yield (percent goal) when there are already critical issues with available water. The implementation involved the use of inverse weighted logistic functions to simulate a bottleneck as follows:

$$y_{ij}={\gamma_{j}\left(\frac{1}{l\left(w_{i}\right)}+\frac{1}{l(r_{i})}+\frac{1}{l\left(n_{i}\right)}\right)}^{-1},$$

where *l* is the logistic function,

$$l\left(w_{i}\right)=\frac{1}{1+\text{exp}\left(-{\beta_{w}(w}_{i}-\alpha_{w}\right))},$$

and *γ*_*j*_ is a block-specific effect.

Principal components analysis was used to summarize information about available water (*w*_*i*_), root health (*r*_*i*_), and nutrient content (*n*_*i*_) (after normalization of the variables). The first principal component was used as an overall summary of each soil health characteristic.

We used a Bayesian approach, in part, to deal with lack of identifiability (different sets of parameters which can make similar predictions). The following information was used to specify the Bayesian model. The *α*′s and *β*′s (for each category) were assumed to have uninformative normal prior distributions with mean 0 and variance 1000. The group-specific effects *γ*_*j*_ were assumed to have a normal prior with mean *γ*_0_ and variance 1000. *γ*_0_ was assumed to be normally distributed with mean 0 and variance 1000. Bayes factors were computed as a measure of evidence for association between the three soil health components. Each soil health component involved the computation of two Bayes factors, one for the *β* parameter and one for the *α* parameter.

## Results and discussion

### Indicators of water availability

**Fig 1:**
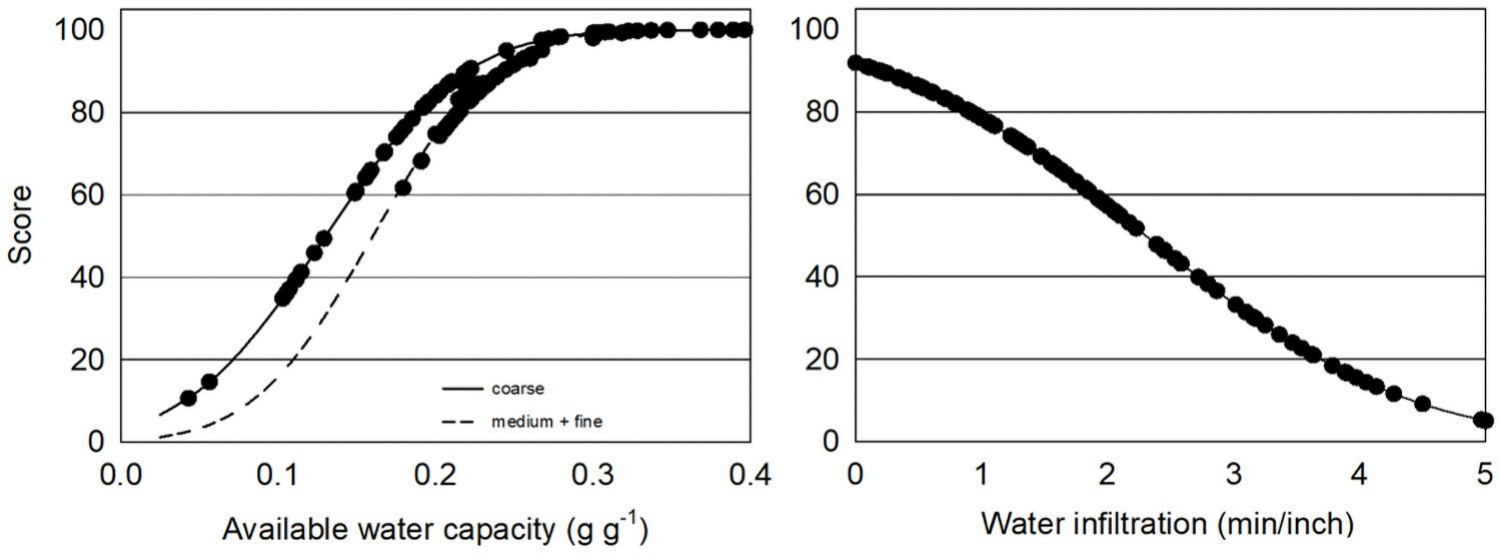
Soil water relations function indicators: Available water capacity (g g^-1^) and water infiltration (min/inch).

Indicators of water availability varied widely across Central Washington apple orchards (*Malus domestica)* where eleven sites had limited water availability. The ability of soils to maintain consistent water availability for orchard crops may be an especially important function in the irrigated West where sufficient, but not excess, water availability is necessary for fruit quality [58–61]. Average rainfall in Central Washington is 178 to 300 mm per year while fruit trees need 3500 to 5000 mm of water per year [37, 62]. Available water capacity (AWC) ranged from 0.2 g g^-1^ in coarse and medium texture soils to 0.3 g g^-1^ in fine textured soils (Table 1; Fig 1). Almost half of the soils sampled had coarse soil texture with an average of 66% sand. AWC is a measure of the porosity of soil in a range important for water retention measured by the amount of water the soil can hold between field capacity and the wilting point [10, 63]. It has been proposed that at AWC > 0.2 g g^-1^ there is no crop limitation, 0.15–0.2 g g^-1^ slight limitation, 0.1–0.15 g g^-1^ moderate, and 0.05–0.1-g g^-1^ severe water limitation [64]. Using these limiting ranges, 5% of sites had AWC indicating severe water limitation and 11% had moderate limitation. While AWC in different soil texture classes is higher in this dataset than the averages found in Cornell’s Comprehensive Assessment Soil Health (CASH) database (Coarse: 0.15 g g^-1^, Medium: 0.21 g g^-1^), the percentage of sites with coarse soil (47%) is higher than the 31% found in Northeastern and Midwestern soils [13]. Water infiltration varied by site with a range of 10 seconds to 5 minutes for 2.5 cm of water to infiltrate. Using a scoring function of the standard distribution (Fig 1), soils where water is able to infiltrate quickly score high. However, in orchards when water quickly drains below crop root zones irrigation needs and nutrient leaching potential increase. A scoring function for water infiltration with an optimum value curve should be designed to account for sufficient but not excess water movement.

**Table 1:** Mean (±SD) for textural distribution and soil health indicators by textural group.

Soil health indicator	Coarse (n = 47)	Medium (n = 45)	Fine (n = 9)
Sand (%)	66.1 ± 12.5	41.4 ± 8.5	35.1 ± 7.4
Silt (%)	27.3 ± 11.6	44.9 ± 11.1	30.9 ± 7.5
Clay (%)	6.5 ± 3.4	13.6 ± 6.2	34.0 ± 4.1
Available water capacity (g g^-1^)	0.20 ± 0.1	0.23 ± 0.0	0.28 ± 0.1
Infiltration (min/inch)	1.7 ± 1.3	2.0 ± 1.5	2.4 ± 1.1
Root health rating apple (0–1)	0.6 ± 0.3	0.6 ± 0.3	0.7 ± 0.2
Root health rating bean (2–9)	4.3 ± 2.0	3.9 ± 1.6	3.0 ± 1.0
*Pratylenchus spp*. (per 500 g)	38.5 ± 43.7	42.7 ± 43.8	45.8 ± 41.0
*Xiphinema spp*. (per 500 g)	0.6 ± 1.7	0.8 ± 3.5	0.0 ± 0.0
OM (%)	2.1 ± 0.9	2.2 ± 0.7	3.8 ± 1.3
Active carbon (ppm)	518.0 ± 201.5	434.6 ± 126.5	517.5 ± 202.0
Bulk density (g cm^-3^)	1.3 ± 0.2	1.3 ± 0.2	1.1 ± 0.2
Water stable aggregates (%)	29.5 ± 18.4	19.1 ± 10.3	26.9 ± 9.2
Surface compaction (kPa)	1110 ± 380	1118 ± 387	1045 ± 622
Subsurface compaction (kPa)	1834 ± 376	1803 ± 483	1728 ± 234
Respiration (mg Co2 g^-1^)	0.5 ± 0.2	0.5 ± 0.2	0.2 ± 0.2
Micro arthropods (m^-2^)	6880.4 ± 14158.8	4106.6 ± 6945.9	14401.2 ± 7300.6
Structure Index (0–100)	36.0 ± 20.2	36.6 ± 24.3	36.8 ± 18.6
PMN (μ N g^-1^ week^-1^)	21.1 ± 33.2	15.1 ± 10.5	6.5 ± 3.2
ACE protein (mg g^-1^)	6.7 ± 3.2	4.3 ± 1.6	4.5 ± 1.8
Enrichment Index (0–100)	62.9 ± 15.9	64.8 ± 18.2	58.6 ± 16.3
pH	7.2 ± 0.5	7.3 ± 0.4	7.0 ± 0.6
P (ppm)	39.5 ± 29.7	33.8 ± 21.8	10.6 ± 6.4
K (ppm)	251.1 ± 140.1	330.2 ± 114.2	306.6 ± 72.3
Ca (ppm)	3881.9 ± 3469.3	3306.6 ± 1955.3	1561.4 ± 358.0
Mg (ppm)	249.3 ± 129.8	344.4 ± 139.6	209.3 ± 54.6
Mn (ppm)	8.0 ± 8.8	16.5 ± 19.8	40.6 ± 17.7
Cu (ppm)	0.2 ± 0.9	0.7 ± 1.6	2.7 ± 0.7
Zn (ppm	13.4 ± 11.8	9.8 ± 14.2	40.7 ± 25.6
Fe (ppm)	10.0 ± 35.1	16.8 ± 35.0	117.5 ± 30.8
CEC (cmolc/kg)	11.0 ± 3.5	14.7 ± 5.3	10.3 ± 2.2

PMN = Potentially Mineralizable N, ACE = Soil Protein, OM = Organic Matter

### Indicators of root health

**Fig 2:**
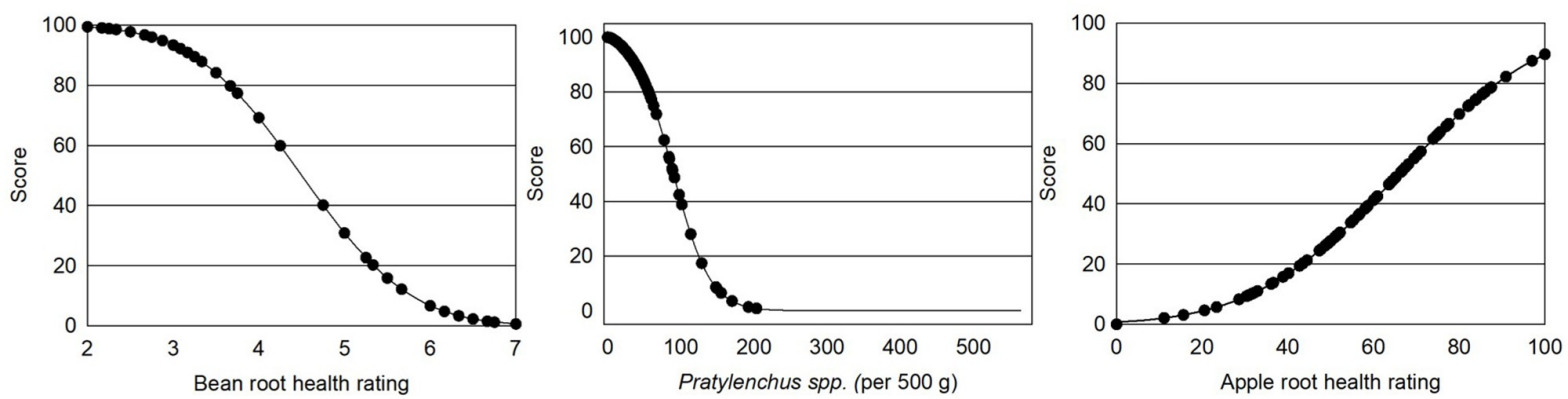
Indicators measuring the root health function of soil including bean root health rating, numbers of *Pratylenchus penetrans* nematodes, and apple root health rating.

Indicators of root health function showed disease potential in 50% of Central Washington apple orchard fields sampled according to apple root health ratings (values <50%), 29% of fields according to bean root health ratings (values 5–9), and 15% based on *Pratylenchus penetrans* nematode counts (Fig 2; Table 1). Plant pathogens *Phytophthora* and *Pythium*, *Ilyonectria robusta*, *Rhizoctonia solani* as well as the lesion nematode *P*. *penetrans* are known to negatively impact growth and production in young apple trees [65–67]. Twenty-nine percent of orchard fields showed moderate damage to advanced decay in bean root health ratings used to detect disease potential for common plant pathogens [54]. In apple root health bioassays, half of the sites had seedlings which grew only 50% as large in unpasteurized soil compared to those in pasteurized soil indicating pressure from soil borne pathogens. Counts of *P*. *penetrans* extracted from bulk soil showed 15 sites where *P*. *penetrans* numbers exceeded 80 per 500 cm^3^, the damage threshold for young trees [68]. In comparison to soils from the Northeast, Midwest and Mid-Atlantic in the CASH database Central Washington orchards had lower root disease potential in medium and fine soils (3.9 and 3.0 WA vs 4.4 and 4.2 CASH) and similar values in coarse textured soils (4.3 WA vs 4.5 CASH) [13]. Most other soil health indices discussed in the literature do not include indicators of root disease potential, important when crop yield and quality are management goals [28, 29, 33].

### Macro, micro-nutrients and pH

**Fig 3:**
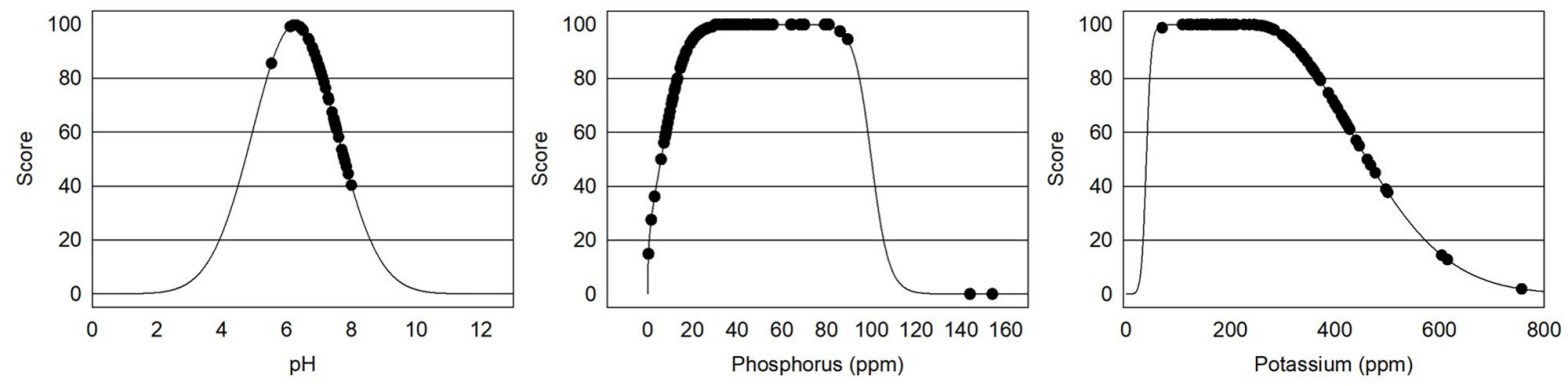
Soil fertility indicators: pH, P and K in 101 Central Washington orchards surveyed.

Central Washington orchards surveyed generally had optimum macro and micronutrient levels (Fig 3). Chemical properties of soil are well established indicators of soil fertility [69, 70]. pH levels in Central Washington orchards surveyed were generally within the optimum range of 6.0 to 7.5 with one site at a limiting level of 5.5 where macronutrients would be less available and twenty-eight sites above 7.5 but below 8.0 [71]. Only 12% of sites had P levels below 10 ppm considered limiting for tree fruit and two sites had excessive levels (>50 ppm). Potassium levels generally were equal to, or greater than, optimum (150–250 ppm) with the exception of four sites that had soil K concentrations of less than 100 ppm and eleven sites were between 100 and 150 ppm. However, forty seven sites had K levels greater than 300 ppm. All sites had Ca and Mg values within optimum ranges and all but two sites received a 100% score for micronutrients (Table 1). Washington orchardists are generally managing nutrient availability adequately using existing soil and tissue testing tools.

### Microbially available nitrogen indicators

**Fig 4:**
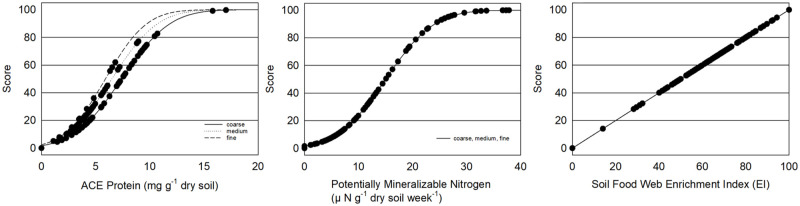
Indicators of microbially available N: ACE protein (mg g^-1^ dry soil), potentially mineralizable N (μ N g^-1^ week^-1^), and soil food web enrichment index for 101 Central Washington orchard field soils.

Measurements of microbially available N in Central Washington orchards showed a range of levels with many orchards where substantial organic N pools should be accounted for when nutrient applications are made. Plant available N from the organic N pool can be estimated from extracted soil proteins, ammonia mineralized during an incubation period, and numbers of soil fauna related to nutrient mineralization [43, 72–74]. Soil quality indices can use mineralizable N values to make crop specific recommendations by subtracting mineralizable N from nutrient requirements [16, 17]. Washington orchards measured had average PMN of 21.1, 15.1 and 6.5 μ N g^-1^ week^-1^ for coarse, medium and fine soils, respectively. ACE soil protein was relatively low with 6.7, 4.3 and 4.5 mg g^-1^ for coarse, medium and fine soils, respectively. The soil food web Enrichment Index varied widely with 82% of sites showing an EI rating of 50 or higher indicating soil fauna with a large capacity to respond to and mineralize N additions (Table 1; Fig 4). High mineralizable N availability in some orchard sites would reduce N application needs. For example, for apples where 80 kg N ha^-1^ is recommended each season to reach a 15 T ha^-1^ yield goal, the average PMN of 21 μ g^-1^ week^-1^ would supply 2.7 kg N ha^-1^ week^-1^ reducing N needs by 55 kg ha^-1^ over the 20 weeks season. Indeed, N applications may reduce fruit quality in these soils. Unfortunately, while extractable organic N fractions are generally positively correlated with mineralizable N, they often only partially explain the variation in mineralizable N and there is disagreement about which test provides more usable information [74, 75].

### Soil structure indicators

**Fig 5:**
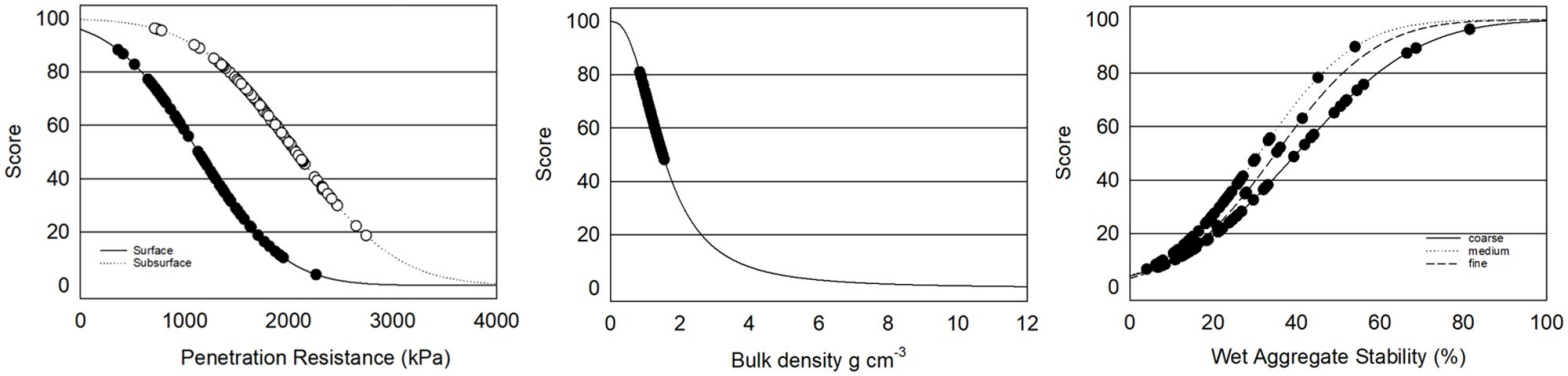
Indicators of soil structure: Penetration resistance, bulk density, and wet aggregate stability in 101 Central Washington orchards field soils.

Soil structure in Central Washington orchards surveyed had moderate to low wet aggregate stability (WAS: average 19% medium, 27% fine and 30% coarse), bulk density (BD) averaging 1.1 to 1.3 g cm^-3^ for fine and medium-coarse soils and moderate surface and subsurface penetration resistance (PR) with the exception of twenty six sites where subsurface PR exceeded 2070 kPa (300 psi) (Table 1; Fig 5). Soil structure and its stability impact the movement and storage of water, aeration, erosion, biological activity and growth of crops [76]. Soils with high BD decrease root development and limit infiltration leading to poor plant growth and potential for run-off [77]. Soil with BD below 1.5 g cm^-3^ can have larger root growth and greater yield [78–82] where soils with BD above 1.8 g cm^-3^ can severely reduce root growth [55]. On average, BD was 1.1 g cm^-3^ in fine texture soils and 1.3 g cm^-3^ in medium and coarse texture soils and lower than levels proposed to impact root growth and yield. Five orchard fields had BD of 1.5 g cm^-3^ indicating a potential limitation in some sites. However, responses can vary among plant species [55] and by soil type and the hypothetical threshold proposed by Raiesi [57] will need to be further evaluated for apple roots. Compaction measured as penetration resistance is considered to limit root growth as well as access to water and nutrients when levels exceed 2070 kPa (300 psi) [83–86]. Twenty-six of the sites had subsurface PR higher than 2070 kPa with five sets of matched pairs where subsurface PR was higher in low yielding orchard sites compared to high yielding sites indicating a potential limiting effect. Average subsurface PR was lower in Washington orchards (fine:1728 kPa, coarse: 1834 kPa) than in the CASH database (fine: 2048 kPa, coarse: 2199) [13]. However, critical levels have been established with annual crops (e.g. maize, peas, barley) and while their relationship to root growth in apples is unknown, tree root growth has been found to decrease exponentially with increasing soil resistance for pine roots [83, 87]. Wet aggregate stability (WAS), held together by roots, fungal hyphae, polysaccharides, clay, metal cations and Ca and Mg carbonates, is considered an indicator of erodibility [76]. Proposed critical levels for WAS include 50–75% slight, 25–50% moderate, 5–25% severe and <5% extreme; and 45–70% slight, 25–45% moderate, 15–25% severe and <15% extreme [33]. WAS in this study averaged 19% for medium, 27% fine and 30% in coarse-textured soils. These values are considerably lower than those in the CASH database for Northeastern and midwestern soils (41–52% average) [13]. However, soil erodibility from low WAS may be less critical in Washington’s perennial apple orchards where the grower standard practice of grass drive rows limits surface soil and water movement.

### Soil biological activity and food web structure

**Fig 6:**
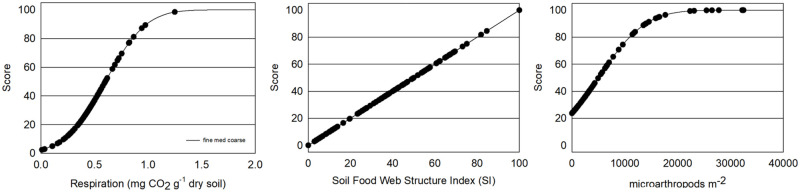
Indicators of soil biological activity and food web community structure: Respiration (mg CO^2^ g^-1^), soil food web structure index (0–100 scale) and micro arthropods (m^-2^) in 101 Central Washington orchards fields.

Indicators of soil biological activity and food web structure were on average moderate too low in this study but were highly variable. Large, active soil biological communities are generally considered beneficial for organic matter and nutrient cycling, protection from plant pathogens and maintenance of soil structure [88–92]. Microbial activity measured by respiration varied from 0.01 to 1.25 mg CO^2^ g^-1^ (Table 1; Fig 6). These respiration levels were generally lower with 78% of samples below the scoring curve average of 0.6 mg CO^2^ g^-1^ [13]. Soil food web structure was also low on average with 78% of sites scoring less than 50% as calculated by the soil food web Structure Index. Micro arthropods including fungal feeding and predatory mites and collembolan in the surface soils were highly variable from 0 to 90,000 counts m^-2^. Indicators of soil food web function can be difficult to interpret due to their dynamic nature in response to temperature, moisture and food resources [23].

### Organic matter measurements

**Fig 7:**
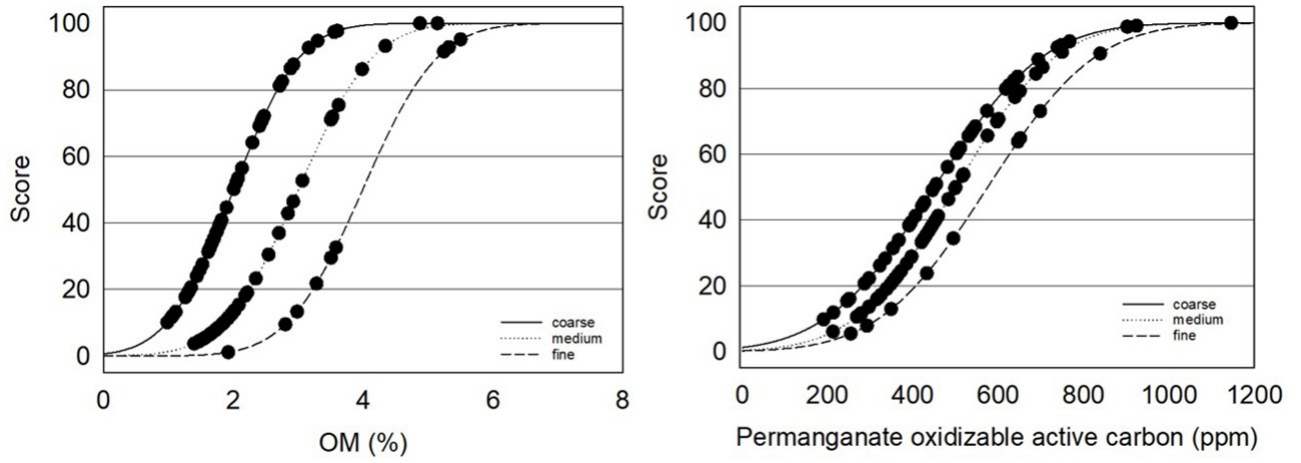
Soil organic matter (OM %) and permanganate oxidizable carbon (POXC ppm) in 101 Central Washington orchards fields.

Washington orchard soils surveyed had a wide range of organic matter, but levels were generally lower than those documented in other regional surveys. Soil organic matter influences multiple soil functions including nutrient cycling and availability, soil carbon accumulation, water holding and soil aggregation and SOM losses can lead to yield reductions [93–96]. Active carbon (e.g. permanganate oxidizable carbon: POXC) composed of the more labile and generally more recently introduced fraction of soil organic carbon is sensitive to management, correlated with microbial biomass, reflects long term carbon sequestration and can be predictive of crop yields [32, 45, 97–100]. Organic matter in Central Washington orchards ranged from 1.0 to 5.5%, with POXC ranging from 191 to 1145 ppm. Fifty seven percent of soils had less than 2% OM and scored less than 50% on the scoring curve for POXC indicating relatively low carbon availability (Table 1, Fig 7). OM in Washington orchards averaged 2.1, 2.2 and 3.8% for coarse, medium and fine textured soils respectively, lower than 3.2, 3.7 and 4.4% OM reported for soils in the CASH database [13]. POXC levels were also lower than in many other studies with an average of 434 ppm in medium textured soils and 517–518 ppm in coarse and fine textured soils compared to medians of 588 ppm (n = 1385) in Culman, Snapp [97] and 486, 531, 608 ppm for coarse, medium and fine textured soils (n = 5767) Fine, van Es [13]. Lower levels of SOC and POXC in this survey compared to those in South Central, Northwest, and Midwest regions are not surprising as SOC is generally lower in lower precipitation areas and in coarse textured soils [101].

### Soil health index minimum dataset

In order to identify a minimum dataset for a soil health index for Central Washington orchards several methods were employed to relate soil factors to management factors important to stakeholders: fruit yield and fruit quality. A lasso regression with cross validation was conducted due to the large number of variables and the small sample size. Respiration, PR, AWC, PMN, and apple root health rating were selected by the model. The model R^2^ was only 0.1135. The results of the model indicate no strong predictors of percent yield goal using a lasso regression.

In order to better understand why we were not identifying certain factors that have documented soil health impacts, we produced plots of the yield outcome against each soil health indicator grouped by block. Using an integration of biological knowledge of the system we looked for trends to see at what thresholds yield trended to decrease between matched pairs (sets of two orchards with matching scion, rootstock and location) as a factor increased or decreased. *Pratylenchus* nematode has a known threshold where 20–70 *Pratylenchus* 500 g^-1^ may cause crop damage and 80 + is likely to damage young trees [68]. All six matched pairs with values over 80 *Pratylenchus* 500 g^-1^ have a downward slope indicating potential reduced yield capacity at high *Pratylenchus* nematode densities. The bean root health rating is on a scale of 1 to 9 where 1 is healthy and values over 4 generally show root damage [54]. In this dataset all the fields with values over 5 have a negative slope where percent yield goal decreases as bean root health rating values increase. AWC of 0.1–0.15 g g^-1^ is thought to create moderate water limitation with AWC less than 0.1 g g^-1^ severely limiting water availability. In exploratory analysis AWC showed negative slopes for matched pairs where low yielding sites had less than 0.15 g g^-1^ AWC. Additionally, in soils with over 70% sand, a downward trend for percent yield goal was apparent.

**Fig 8:**
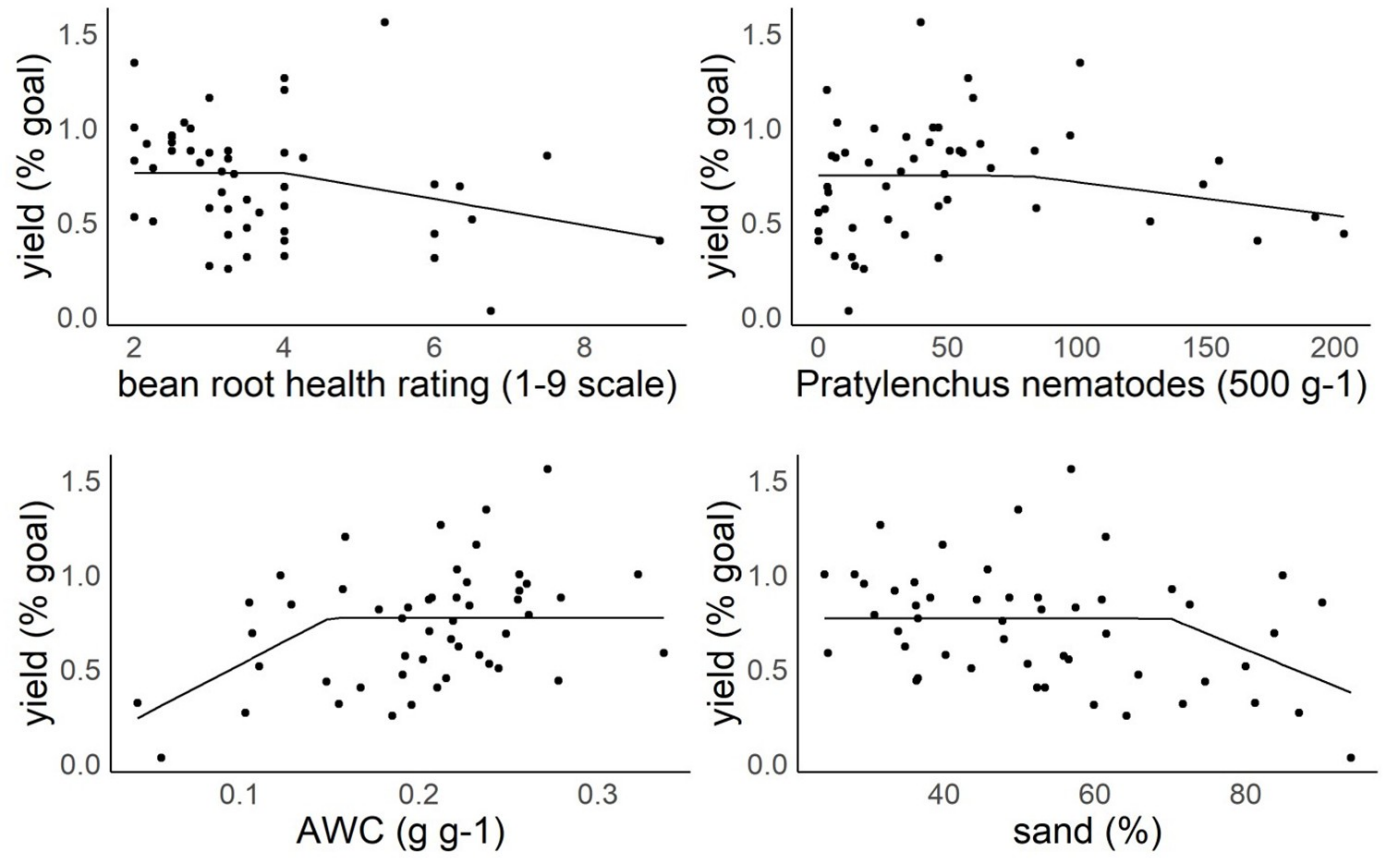
Linear mixed effects model Pratylenchus nematodes (500 g^-1^), bean root health rating (1–9 scale), AWC (g g^-1^), sand (%) and yield (% goal). AWC = available water capacity.

We then used a linear mixed effects model to characterize the association between yield (percent goal) and each of the selected soil health factors which showed strong trends in exploratory analysis. Identification using this methodology is subject to selection bias (e.g., you will find one plot that appears to have an effect just by chance because you are screening across a number of plots) and as such, p-values reported from subsequent tests should be considered exploratory. *Pratylenchus* nematode with a threshold of 80 and bean root health rating with a threshold of 4 had consistent but not significant yield (percent goal) models (P = 0.24, P = 0.08; Fig 8, S2 Table), with similar results for packout (P = 0.19, P = 0.17). AWC with a threshold of 0.15 and % sand with a threshold of 70% had significant yield (percent goal) models (P = 0.009; P = 0.03; Fig 8), with non significant results for packout (P = 0.09; P = 0.20).

Principal components analysis (PCA) was used to reduce the number of variables. 92 percent of the variation in AWC and % sand was explained by the first principal component (after normalization of the variables). Thirty nine percent of the variation in the apple root health rating, bean root health rating, and *Pratylenchus* nematodes was explained by the first principal component (after normalization of the variables). Fifty percent of the variation in pH, P, K was explained by the first principal component. While root health rating (apple and bean) predicted a relatively small amount of the variation, these factors are included due to well established impact on yield and fruit quality [65–67]. Loading factors were AWC: 0.71 and sand: -0.71 for principal component *Water Availability*; apple root health rating 0.66, bean root health rating -0.55, and *Pratylenchus* nematode -0.50 for principal component *Root Health*, and pH 0.28, P 0.64 and K 0.72 for principal component *Nutrient Availability*.

A nonlinear Bayesian model was computed as a measure of evidence for association between soil health components and tree fruit productivity. Bayes factors < 1 (on either parameter, *α* or *β*) are interpreted as evidence for an association between the soil health factor and productivity. For water, the yield (percent goal) Bayes factor was 0.20 for *β*_*w*_ and 0.68 for α_w_ and the pack-out (boxes of packed fruit) Bayes factor was 1.07 for *β*_*w*_ and 0.91 for α_w_ (S3 Table). For root health, the yield (percent goal) Bayes factor was 2.78 for *β*_*r*_ and 0.65 for α_r_, and the pack-out Bayes factor was 1.00 for *β*_*r*_ and 0.97 for α_r_. For fertility, the yield (percent goal) Bayes factor was 1.14 for *β*_*f*_ and for 0.76 α_f_ and the pack-out Bayes factor was 1.02 for *β*_*f*_ and 0.92 for α_f_.

The Bayesian model was run for 10,000,000 iterations; however, convergence was still questionable. More informative priors are needed to better identify the model and allow for convergence in a reasonable timeframe. Therefore, Bayes factors should be considered with some skepticism, and we feel that results from the linear mixed model are more informative. Future improvements to the model could involve more accurate specification of the prior α values using knowledge about reasonable ranges of the soil health parameters. Overall, we feel that the Bayesian model will better serve to represent nonlinear interdependence of the soil health variables and outcomes when priors can be adequately specified.

The current study suggests that the minimum dataset of soil health indicators for Central Washington orchards should include measurements of water availability (AWC, % sand) and of root health (bean root health rating, *Pratylenchus* nematodes) as well as fertility indicators to meet stakeholder management goals. In contrast other studies of annual crops identified soil health factors related to soil C. Active carbon, and total soil carbon were related to yield in four studies [22, 30–32] as well as WSA [31, 33] and respiration [30] in some studies. Soil health indices which focus on environmental processes and response to management [10, 17] or median values from regional datasets [18, 19, 21] also include soil carbon, mineralizable N, BD, and fertility indicators. However, with the exception of Idowu [27] root health indicators were not measured in most of these studies consequently ignoring the potential importance of root disease pressure from soils [4, 7, 10, 18, 28, 33]. To our knowledge the only other soil health assessment tool for perennial crops in the irrigated west is from Glover, Reganold, and Andrews [29]. The approach taken is laudable as it bases scores on empirically derived baseline and optimal values for individual indicators and combines a combination of environmentally related and production related goals. However, included indicators were not selected based on appropriateness to measure relevant management goals.

## Conclusions

Indicators measured in Central Washington orchards had a wide range but with generally lower organic matter, lower available water capacity, higher percent sand and lower wet aggregate stability than Midwest, Mid-Atlantic and Northeastern (United States) soils measured in other studies. The minimum dataset of soil health indicators for Central Washington orchards should include measurements of water availability (AWC, % sand) and of root health (bean root health rating, *Pratylenchus* nematodes) as well as fertility indicators to meet stakeholder management goals. High levels of mineralizable N in some orchards indicate the need to include a measurement of organic N availability in the minimum data set. With more than 25% of surveyed orchards with high subsurface PR values, a measurement of compaction should be included. While OM and POXC were not correlated with the stakeholder management goal of productivity, soil organic matter influences multiple soil functions including microbial activity, nutrient cycling, soil carbon accumulation and water relations, and as such should be included in the minimum dataset as indicators of environmental health.

## Supporting information

S1 Table Supplementary data: Descriptions of study sites. (DOCX)

S2 Table Supplementary data from individual orchards for yield and packouts for 32 orchards sampled from 2017–2019. (DOCX)

S3 Table Maximum a posteriori estimates of the parameters of the Bayesian model as well as the variance of the block specific effects. (DOCX)

S1 Fig Map of study sites in Central Washington, United States. (TIF)
