# Supplementary material for: Soil health indicators for Central Washington orchards
Source: PLoS One. 2021 Oct 28;16(10):e0258991. doi: 10.1371/journal.pone.0258991 (PMC8553132; doi:10.1371/journal.pone.0258991)
Supplement: S1 Table — (DOCX) [file pone.0258991.s001.docx]

**S1. Table: Supplementary data: descriptions of study sites.**

| year | block | site | scion | rootstock | tree age^Ϫ^ | OM Mg ha^-1¥^ | soil name | soil texture | trees ha^-1^ | tree training system | orchard floor management |
| --- | --- | --- | --- | --- | --- | --- | --- | --- | --- | --- | --- |
| 2016 | B | 1 | Gala | M9 | 12 | ND | Kennewick | sandy loam | 3262 | Central leader | mow-blow |
| 2016 | B | 2 | Red Delicious | M106 | 12 | ND | Kennewick | sandy loam | ND | Central leader | mow-blow |
| 2016 | G | 3 | Honeycrisp | seedling | 8 | ND | Colville, Okanogan | silt loam | 298 | Central leader | mow |
| 2016 | G | 4 | Honeycrisp | seedling | 8 | ND | Pogue | sandy loam | 332 | Central leader | mow |
| 2016 | Th | 7 | Red Delicious | ND | 15 | ND | Tonasket | silt loam | 1794 | Central leader | herbicide |
| 2016 | Th | 8 | Red Delicious | ND | 15 | ND | Tonasket | silt loam | 1794 | Central leader | herbicide |
| 2016 | F | 9 | Gala | M9.337 | 20 | ND | Thowson | fine sandy loam | 2990 | Tall spindle | cultivated |
| 2016 | F | 10 | Pinata | M9.337 | 10 | ND | Thowson | fine sandy loam | 1595 | Tall spindle | cultivated |
| 2016 | R | 11 | Gala | ND | 10 | ND | Adkins | loam | ND | V trellis | mow blow |
| 2016 | R | 12 | Gala | ND | 10 | ND | Adkins | sandy loam | ND | V trellis | mow blow |
| 2016 | B | 13 | Red Delicious | ND | 10 | ND | Adkins | sandy loam | ND | V trellis | mow blow |
| 2016 | M | 14 | Red Delicious | ND | ND | ND | Warden | silt loam | 2990 | Tall spindle | mow blow |
| 2016 | M | 15 | Red Delicious | ND | ND | ND | Warden | silt loam | 2990 | Tall spindle | mow blow |
| 2016 | MJ | 16 | Gala | ND | ND | ND | Neppel | silt loam | 2990 | Tall spindle | mow blow |
| 2016 | Cw | 17 | Nicoter gala | M9 | ND | ND | Sagehill, Taunton | loam | 4306 | Tall spindle | herbicide |
| 2016 | Cw | 18 | Nicoter gala | M9 | ND | ND | Sagehill, Taunton | loam | 4306 | Tall spindle | herbicide |
| 2016 | WA | 19 | WA38 | M9 | 4 | ND | Shano | silt loam | 2990 | Tall spindle | herbicide |
| 2016 | WA | 20 | WA38 | M9 | 4 | ND | Pogue, Quincy | loamy sand | 2990 | Tall spindle | herbicide |
| 2016 | P | 21 | Winsap | G40 | 4 | ND | Quincy | sand | 5382 | Tall spindle | cultivated |
| 2016 | P | 22 | W2 | G40 | 4 | ND | Quincy | sand | 5980 | Tall spindle | cultivated |
| 2016 | Br | 27 | Gala | M26 | 20 | ND | Chelan | sandy loam | 1621 | Tall spindle | cultivated |
| 2016 | Br | 28 | Gala | M27 | 10 | ND | Suplee | loamy sand | 2928 | Tall spindle | cultivated |
| 2016 | SR | 29 | Honeycrisp | M9 | 4 | ND | Thowson | loam | 3588 | Tall spindle | herbicide |
| 2016 | SR | 30 | Honeycrisp | M9 | 4 | ND | Thowson | loam | 3588 | Tall spindle | herbicide |
| 2016 | SR | 31 | Juicy | M9 | 4 | ND | Thowson | loam | 3588 | Tall spindle | herbicide |
| 2017 | Bk | 32 | Honeycrisp | M9.Nic29, M9.337 | 5 | 0 | Scoon | silt loam | 2990 | Tall spindle | herbicide |
| 2017 | Bk | 33 | Honeycrisp | M9.Nic29, M9.337 | 5 | 0 | Scoon | silt loam | 2990 | Tall spindle | herbicide |
| 2017 | Cl | 34 | Gala | M9 | 7 | 29 | Entiat | sandy loam | 3262 | Tall spindle | cultivated |
| 2017 | Cl | 35 | Gala | M9 | 7 | 29 | Pogue | sandy loam | 3262 | Tall spindle | cultivated |
| 2017 | SF | 36 | Red Delicious | M106 | 22 | 0 | Burke, Willis | silt loam | 664 | Central leader | herbicide |
| 2017 | SF | 37 | Red Delicious | M106 | 22 | 0 | Burke, Willis | silt loam | 664 | Central leader | herbicide |
| 2017 | Hou | 38 | Granny Smith | M9.337 | 6 | 10 | Kennewick | coarse silt | 3758 | Tall spindle | mow-blow-herbicide |
| 2017 | Hou | 39 | Granny Smith | M9.337 | 6 | 10 | Kennewick | coarse silt | 3758 | Tall spindle | mow-blow-herbicide |
| 2017 | AB | 40 | Honeycrisp | M9.337 | 10 | 0 | Cleman | fine sandy loam | 2242 | Fruiting wall | herbicide |
| 2017 | AB | 41 | Honeycrisp | M9.337 | 10 | 0 | Cleman | fine sandy loam | 2242 | Fruiting wall | herbicide |
| 2017 | AB2 | 42 | Honeycrisp | M9.337 | 6 | 0 | Cleman | fine sandy loam | 3194 | Fruiting wall | herbicide |
| 2017 | AB2 | 43 | Honeycrisp | M9.337 | 6 | 0 | Cleman | fine sandy loam | 3194 | Fruiting wall | herbicide |
| 2017 | Wi | 44 | Granny Smith | M106 | 8 | 0 | Chelan | gravelly sandy loam | 2242 | Central leader | herbicide |
| 2017 | Wi | 45 | Granny Smith | M106 | 8 | 0 | Chelan | gravelly sandy loam | 2242 | Central leader | herbicide |
| 2017 | F | 46 | Gala | M9.337 | 4 | 20 | Tonasket | silt loam | 3588 | Tall spindle | herbicide |
| 2017 | F | 47 | Gala | M9.337 | 4 | 20 | Tonasket | silt loam | 3588 | Tall spindle | herbicide |
| 2017 | KG | 48 | Granny Smith | M106 | 9 | 4 | Pogue | fine sandy loam | 664 | Central leader | cultivated |
| 2017 | KG | 49 | Granny Smith | M26 | 9 | 4 | Pogue | fine sany loam | 1538 | Central leader | cultivated |
| 2018 | O | 50 | Gala | M9.337 | 10 | 0 | Warden | silt loam | 1350 | Central leader | herbicide |
| 2018 | O | 51 | Gala | M9.337 | 10 | 0 | Warden | silt loam | 1350 | Central leader | herbicide |
| 2018 | Zi | 52 | Gala | M26 | 2 | 67 | Kennewick, Taunton | loam | 1256 | Central leader | mowblow |
| 2018 | Zi | 53 | Gala | M26 | 21 | 11 | Taunton | loam | 1621 | Central leader | mowblow |
| 2018 | K | 54 | Gala | M7 | 18 | 26 | Warden | fine sandy loam | 823 | Grafted three leader | herbicide |
| 2018 | K | 55 | Gala | M7 | 19 | 0 | Loagy | silt loam | 2197 | Grafted three leader | herbicide |
| 2018 | WAF | 56 | Honeycrisp | M9.337 | 9 | 34 | Taunton | sandy loam | 2928 | Spindle | herbicide |
| 2018 | WAF | 57 | Honeycrisp | M9.337 | 9 | 34 | Taunton, Scoon | sandy loam | 2928 | Spindle | herbicide |
| 2018 | Rb | 58 | Gala | M9.Nic29 | 11 | 0 | Cashmont | sandy loam | 2928 | Spindle | herbicide |
| 2018 | Rb | 59 | Gala | M9.Nic29 | 11 | 0 | Cashmont | sandy loam | 2928 | Spindle | herbicide |
| 2018 | GO | 60 | Envy | M9.Nic29 | 3 | 27 | Chelan | loam | 2928 | V trellis | herbicide |
| 2018 | GO | 61 | Envy | M9.Nic29 | 3 | 27 | Chelan | sandy loam | 2928 | V trellis | herbicide |
| 2018 | T | 62 | Fugi | M111 | 5 | 3 | Pogue | sandy loam | 1025 | Grafted four leader | herbicide |
| 2018 | T | 63 | Fugi | M111 | 12 | 0 | Pogue | sandy loam | 1266 | Grafted four leader | mowed |
| 2018 | AR | 64 | Honeycrisp | seedling | 20 | 13 | Peshastin | sandy loam | 1121 | Grafted three leader | herbicide |
| 2018 | AR | 65 | Honeycrisp | seedling | 20 | 13 | Peshastin | sandy loam | 1121 | Grafted three leader | herbicide |
| 2018 | Zi2 | 66 | Gala | M26 | 22 | 2 | Kennewick | silt loam | 1256 | Central leader | mowblow |
| 2018 | Zi2 | 67 | Gala | M26 | 22 | 0 | Kennewick, Taunton | loam | 1621 | Central leader | herbicide |
| 2019 | KMO | 68 | Pinata | ND | 6 | 179 | Colockum, Zen | loam | 968 | Tall spindle | cultivated |
| 2019 | KMO | 69 | Pinata | ND | 6 | 179 | Zen | sandy loam | 968 | Tall spindle | cultivated |
| 2019 | S | 70 | Honeycrisp | M9 Pajam 2® | 10 | 0 | Shano | silt loam | 2928 | Tall spindle | cultivated |
| 2019 | S | 71 | Honeycrisp | M9 Pajam 2® | 9 | 0 | Shano | silt loam | 2928 | Tall spindle | cultivated |
| 2019 | DS | 72 | Fugi | M9 | 11 | 118 | Burbank | loamy sand | 4392 | Tall spindle | cultivated |
| 2019 | DS | 72 | Fugi | M9 | 11 | 118 | Burbank | loamy sand | 4392 | Tall spindle | cultivated |
| 2019 | DS | 73 | Fugi | M9 | 11 | 118 | Burbank | loamy sand | 4392 | Tall spindle | cultivated |
| 2019 | CS | 74 | Fugi | M9.337 | 15 | 0 | Taunton, Scoon | sandy loam | 4392 | Tall spindle | cultivated |
| 2019 | CS | 75 | Fugi | M9.337 | 15 | 0 | Taunton, Scoon | sandy loam | 4392 | Tall spindle | cultivated |
| 2019 | Gil | 76 | Gala | M9 | 6 | ND | Burke, Shano, Kiona | loam | 4392 | V trellis | herbicide |
| 2019 | Gil | 77 | Gala | M9 | 6 | 0 | Kiona | sandy loam | 4392 | V trellis | herbicide |
| 2019 | Gil2 | 78 | Granny Smith | M9.337 | 5 | 0 | Burke, Shano | sandy loam | 5382 | V trellis | herbicide |
| 2019 | Gil2 | 79 | Granny Smith | M9.337 | 6 | 0 | Shano | sandy loam | 5382 | V trellis | herbicide |
| 2019 | Gil3 | 80 | Red Delicious | M26 | 25 | 94 | Shano | sandy loam | 747 | Central leader | mowed |
| 2019 | Gil3 | 81 | Red Delicious | M26 | 25 | 0 | Shano | sandy loam | 747 | Central leader | herbicide |
| 2019 | Zi3 | 82 | Honeycrisp | M26 | 4 | 38 | Kennewick | silt loam | 4392 | Central leader | herbicide |
| 2019 | Zi3 | 83 | Honeycrisp | M26 | 4 | 38 | Taunton | sandy loam | 1505 | Central leader | herbicide |
| 2019 | Va | 84 | Honeycrisp | M106 | 5 | 9 | Warden | silt loam | 4893 | Tall spindle | herbicide |
| 2019 | Va | 85 | Honeycrisp | M106 | 5 | 13 | Hezel, Quincy | loamy sand | 4893 | Tall spindle | herbicide |
| 2019 | Ob | 86 | Fugi | M9.337 | 9 | 0 | Warden | silt loam | 2990 | Tall spindle | herbicide |
| 2019 | Ob | 87 | Fugi | M9.337 | 9 | 11 | Warden | silt loam | 2990 | Tall spindle | cultivated |
| 2019 | Al | 88 | Gala | M9 | 8 | 224 | Burbank, Quincy | loamy sand | 3514 | Tall spindle | cultivated |
| 2019 | Al | 89 | Gala | M9 | 8 | 224 | Burbank, Quincy | sand | 3514 | Tall spindle | cultivated |
| 2019 | H | 90 | Ambrosia | B.9 | 13 | 0 | Scoon | silt loam | 2990 | Tall spindle | cultivated |
| 2019 | H | 91 | Ambrosia | B.9 | 13 | 20 | Scoon | silt loam | 2990 | Tall spindle | cultivated |
| 2019 | SRO | 92 | Gala | M9.337 | 12 | 22 | Pogue | sandy loam | 2990 | Tall spindle | mow-blow-cultivated |
| 2019 | SRO | 93 | Gala | M9.337 | 12 | 33 | Pogue | sandy loam | 2990 | Tall spindle | mow-blow-cultivated |
| 2019 | SRO | 94 | Gala | M9.337 | 12 | 17 | Pogue | sandy loam | 2990 | Tall spindle | mow-blow-cultivated |
| 2019 | SRO | 95 | Gala | M9.337 | 12 | 9 | Pogue | sandy loam | 2990 | Tall spindle | mow-blow-herbicide |
| 2019 | SRO | 96 | Gala | M9.337 | 12 | 0 | Pogue | sandy loam | 2990 | Tall spindle | fabric mulch |
| 2019 | SRO | 97 | Gala | M9.337 | 12 | 151 | Pogue | sandy loam | 2990 | Tall spindle | woodchip mulch |
| 2019 | SRO | 98 | Gala | M9.337 | 12 | 0 | Pogue | sandy loam | 2990 | Tall spindle | herbicide |
| 2019 | Gil4 | 100 | Gala | M26 | 23 | ND | Gorskel-Harwood | loam | 4306 | Tall spindle | cultivated |
| 2019 | Gil4 | 101 | Gala | M7 | 23 | ND | Harwood | loam | 4306 | Tall spindle | cultivated |
| 2019 | Zi4 | 102 | Honeycrisp | M26 | 4 | 22 | Kennewick | silt loam | 4140 | Central leader | herbicide |
| 2019 | Zi4 | 103 | Honeycrisp | M26 | 4 | 22 | Taunton | sandy loam | 1345 | Central leader | herbicide |

M = Malling, G = Geneva, B= Budagovsky, ND = not determined, mow-blow = mulching technique whereby the grass or other cover crop in the drive row is mowed and blown into the tree row.

^¥^Total organic matter additions including compost, mulch, or grass clippings over the life of the orchard planting until soil sampling date.

^Ϫ^Tree age at date of soil sampling calculated as years in the orchard and not including years in the nursery.

^ξ^ Orchard plots included in analysis with yield included: P21, P22, Br27, Br28, Cl34, Cl35, SF36, SF37, H38, H39, KG48, KG49, O50, O51, Z52, Z53, K54, K55, WA56, WA57, Rb58, Rb59, AR64, AR65, Zi66, Zi67, KMO68, KMO69, S70, S71, Gil76, Gil77, Gil78, Gil79, Zi82, Zi83, Va84, VA85, Ob86, Ob87, Al88, Al89, H90, H91, SRO97, SRO98, Gil4100, Gil4101, Zi102, Zi103. Inclusion was based on plots for which yield data was available, pairs had sufficiently similar site characteristics and sufficient irrigation was applied based on field measurements.
