# Supplementary material for: Soil health indicators for Central Washington orchards
Source: PLoS One. 2021 Oct 28;16(10):e0258991. doi: 10.1371/journal.pone.0258991 (PMC8553132; doi:10.1371/journal.pone.0258991)
Supplement: S2 Table — (DOCX) [file pone.0258991.s002.docx]

**S2. Table Supplementary data from individual orchards for yield and packouts for 32 orchards sampled from 2017-2019.**

| **Year** | **Orchard** | **Cultivar** | **Crop Load (fruit cm^-2^ TCSA)** | **Fruit Tree^-1^** | **Mean Fruit Weight** | **Yield Tree^-1^**  **(kg)** | **Trees ha^-1^** | **Yield**  **(T ha^-1^)** | **% cull** | **Packed boxes Ha^-1^** |
| --- | --- | --- | --- | --- | --- | --- | --- | --- | --- | --- |
| 2017 | BR27+ | Gala | 1.76 | 270 | 176 | 47.4 | 1621 | 76.8 | 6.7 | 3271 |
| 2017 | BR28- | Gala | 3.51 | 49 | 175 | 8.7 | 2928 | 25.3 | 18.3 | 1043 |
| 2017 | AB42+ | Honeycrisp | 3.77 | 87 | 161 | 14.0 | 3194 | 44.7 | 1.7 | 1358 |
| 2017 | AB43- | Honeycrisp | 5.47 | 60 | 179 | 10.7 | 3194 | 34.1 | 1.7 | 1928 |
| 2017 | AB40+ | Honeycrisp | 6.06 | 46 | 260 | 12.0 | 2242 | 26.9 | 1.7 | 1373 |
| 2017 | AB41- | Honeycrisp | 7.73 | 72 | 217 | 15.5 | 2242 | 34.8 | 1.7 | 1881 |
| 2017 | KG48+ | Granny Smith | 2.80 | 421 | 126 | 53.0 | 664 | 34.5 | 5.0 | 1806 |
| 2017 | KG49- | Granny Smith | 0.77 | 71 | 179 | 12.7 | 1538 | 19.1 | 1.7 | 1047 |
| 2017 | H38+ | Granny Smith | 8.68 | 120 | 169 | 20.3 | 3758 | 76.2 | 0.0 | 2479 |
| 2017 | H39- | Granny Smith | 8.28 | 110 | 174 | 19.1 | 3758 | 72.0 | 16.7 | 1587 |
| 2018 | z52+ | Gala | 6.40 | 441 | 144 | 63.5 | 1256 | 79.7 | 1.7 | 3951 |
| 2018 | Z53- | Gala | 4.45 | 137 | 153 | 20.9 | 1621 | 33.8 | 6.7 | 1500 |
| 2018 | Z66+ | Gala | 3.16 | 332 | 141 | 46.8 | 1256 | 58.8 | 1.7 | 2976 |
| 2018 | Z67- | Gala | 2.10 | 133 | 151 | 20.0 | 1621 | 32.4 | 16.7 | 1433 |
| 2018 | RB58+ | Gala | 2.61 | 143 | 163 | 23.4 | 2928 | 68.5 | 0.0 | 3722 |
| 2018 | RB59- | Gala | 3.52 | 91 | 204 | 18.6 | 2928 | 54.4 | 3.3 | 2283 |
| 2018 | K54+ | Gala | 3.84 | 474 | 152 | 72.1 | 823 | 59.4 | 3.3 | 3318 |
| 2018 | K55- | Gala | 4.35 | 125 | 190 | 23.6 | 2197 | 51.9 | 3.3 | 2307 |
| 2018 | WA56+ | Honeycrisp | 2.55 | 121 | 235 | 28.4 | 2928 | 83.1 | 10.4 | 3613 |
| 2018 | WA57- | Honeycrisp | 3.68 | 77 | 258 | 19.9 | 2928 | 58.2 | 29.2 | 1996 |
| 2018 | O50+ | Gala | 2.98 | 237 | 178 | 42.1 | 1350 | 56.9 | 1.7 | 2518 |
| 2018 | O51- | Gala | 4.51 | 221 | 174 | 38.4 | 1350 | 51.9 | 1.7 | 2352 |
| 2019 | Al88+ | Gala | 6.59 | 121 | 185 | 22.4 | 3514 | 78.8 | 1.7 | 3694 |
| 2019 | Al89- | Gala | 5.26 | 53 | 113 | 6.0 | 3514 | 21.1 | 20.0 | 913 |
| 2019 | Gil76+ | Gala | 9.31 | 92 | 133 | 12.2 | 4392 | 53.7 | 26.7 | 1972 |
| 2019 | Gil77- | Gala | 7.99 | 44 | 139 | 6.1 | 4392 | 26.9 | 13.3 | 1116 |
| 2019 | S70+ | Honeycrisp | 4.72 | 139 | 194 | 27.0 | 2928 | 79.0 | 0.0 | 5082 |
| 2019 | S71- | Honeycrisp | 5.00 | 27 | 232 | 6.3 | 2928 | 18.4 | 8.6 | 820 |
| 2019 | Zi82+ | Honeycrisp | 8.74 | 75 | 226 | 16.9 | 4392 | 74.4 | 10.4 | 3685 |
| 2019 | Zi83- | Honeycrisp | 3.61 | 79 | 216 | 17.1 | 1505 | 25.7 | 27.3 | 981 |
| 2019 | KMO68+ | Pinata | 5.65 | 352 | 202 | 71.2 | 968 | 68.9 | 10.0 | 3481 |
| 2019 | KMO69- | Pinata | 4.21 | 233 | 216 | 50.2 | 968 | 48.6 | 8.3 | 2198 |
