# Supplementary material for: Soil health indicators for Central Washington orchards
Source: PLoS One. 2021 Oct 28;16(10):e0258991. doi: 10.1371/journal.pone.0258991 (PMC8553132; doi:10.1371/journal.pone.0258991)
Supplement: S3 Table — (DOCX) [file pone.0258991.s003.docx]

**S3. Table: Maximum a posteriori estimates of the parameters of the Bayesian model as well as the variance of the block specific effects.** The hat indicates the maximum a posterior estimate, and SD indicates the standard deviation of the posterior distribution.

| **Soil Health Variable** | $\hat{\boldsymbol{\beta}}\boldsymbol{(SD)}$ | $\hat{\boldsymbol{\alpha}}\boldsymbol{(SD)}$ | ${\hat{\boldsymbol{\gamma}}}_{\boldsymbol{0}}$ $\boldsymbol{(SD)}$ | $\hat{\boldsymbol{\sigma}}$ **(SD)** | $\boldsymbol{Var}\mathbf{(}\hat{\boldsymbol{\gamma}_{\boldsymbol{i}}}\boldsymbol{)}$ |
| --- | --- | --- | --- | --- | --- |
| Water | 0.163 (0.080) | 24.5 (10.4) | 87.0 (17.3) | 0.267 (0.036) | 305.0 |
| Root | 0.481 (30.4) | 13.3 (32.1) |  |  |  |
| Fertility | 1.32 (31.0) | -12.7 (32.4) |  |  |  |
