# Supplementary figures and images for: Soil health indicators for Central Washington orchards
Source: PLoS One. 2021 Oct 28;16(10):e0258991. doi: 10.1371/journal.pone.0258991 (PMC8553132; doi:10.1371/journal.pone.0258991)

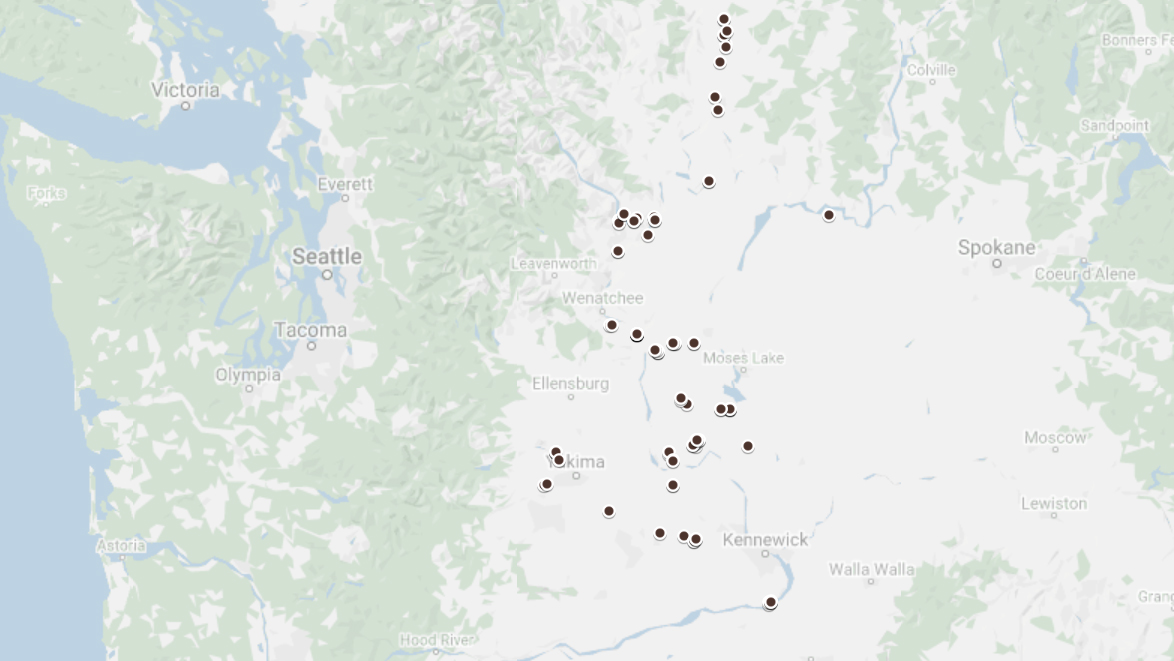

Supplement: S1 Fig — (TIF) [file pone.0258991.s004.tif]
